# Identification and validation of multiple cell surface markers of clinical-grade adipose-derived mesenchymal stromal cells as novel release criteria for good manufacturing practice-compliant production

**DOI:** 10.1186/s13287-016-0370-8

**Published:** 2016-08-11

**Authors:** Emily T. Camilleri, Michael P. Gustafson, Amel Dudakovic, Scott M. Riester, Catalina Galeano Garces, Christopher R. Paradise, Hideki Takai, Marcel Karperien, Simon Cool, Hee-Jeong Im Sampen, A. Noelle Larson, Wenchun Qu, Jay Smith, Allan B. Dietz, Andre J. van Wijnen

**Affiliations:** 1 Department of Orthopedic Surgery, Mayo Clinic, Rochester, MN USA; 2 Department of Laboratory Medicine & Pathology, Mayo Clinic, Rochester, MN USA; 3 Department of Periodontology, Nihon University School of Dentistry at Matsudo, Chiba, Japan; 4 Department of Developmental Bioengineering, University of Twente, Enschede, The Netherlands; 5 Department of Tissue Regeneration, University of Twente, Enschede, The Netherlands; 6 Institute of Medical Biology, Agency for Science, Technology and Research, Singapore, Singapore; 7 Department of Orthopaedic Surgery, Yong Loo Lin School of Medicine, National University of Singapore, Singapore, Singapore; 8 Department of Biochemistry, Rush University Medical Center, Chicago, IL USA; 9 Department of Orthopedic Surgery, Rush University Medical Center, Chicago, IL USA; 10 Department of Internal Medicine, Section of Rheumatology, Rush University Medical Center, Chicago, IL USA; 11 Jesse Brown VA Medical Center, Chicago, IL USA; 12 Department of Physical Medicine and Rehabilitation, Division of Pain Medicine, Mayo Clinic, Rochester, MN USA; 13 Department of Physical Medicine and Rehabilitation, Mayo Clinic, Rochester, MN USA; 14 Department of Radiology, Mayo Clinic, Rochester, MN USA; 15 Department of Anatomy, Mayo Clinic, Rochester, MN USA; 16 Department of Biochemistry and Molecular Biology, Mayo Clinic, Rochester, MN USA

**Keywords:** Adipose-derived mesenchymal stromal cells, RNA-sequencing, Flow cytometry, Release criteria, CD markers, Human platelet lysate, Manufacturing

## Abstract

**Background:**

Clinical translation of mesenchymal stromal cells (MSCs) necessitates basic characterization of the cell product since variability in biological source and processing of MSCs may impact therapeutic outcomes. Although expression of classical cell surface markers (e.g., CD90, CD73, CD105, and CD44) is used to define MSCs, identification of functionally relevant cell surface markers would provide more robust release criteria and options for quality control. In addition, cell surface expression may distinguish between MSCs from different sources, including bone marrow-derived MSCs and clinical-grade adipose-derived MSCs (AMSCs) grown in human platelet lysate (hPL).

**Methods:**

In this work we utilized quantitative PCR, flow cytometry, and RNA-sequencing to characterize AMSCs grown in hPL and validated non-classical markers in 15 clinical-grade donors.

**Results:**

We characterized the surface marker transcriptome of AMSCs, validated the expression of classical markers, and identified nine non-classical markers (i.e., CD36, CD163, CD271, CD200, CD273, CD274, CD146, CD248, and CD140B) that may potentially discriminate AMSCs from other cell types. More importantly, these markers exhibit variability in cell surface expression among different cell isolates from a diverse cohort of donors, including freshly prepared, previously frozen, or proliferative state AMSCs and may be informative when manufacturing cells.

**Conclusions:**

Our study establishes that clinical-grade AMSCs expanded in hPL represent a homogeneous cell culture population according to classical markers,. Additionally, we validated new biomarkers for further AMSC characterization that may provide novel information guiding the development of new release criteria.

**Clinical trials:**

Use of Autologous Bone Marrow Aspirate Concentrate in Painful Knee Osteoarthritis (BMAC): Clinicaltrials.gov NCT01931007. Registered August 26, 2013.

MSC for Occlusive Disease of the Kidney: Clinicaltrials.gov NCT01840540. Registered April 23, 2013.

Mesenchymal Stem Cell Therapy in Multiple System Atrophy: Clinicaltrials.gov NCT02315027. Registered October 31, 2014.

Efficacy and Safety of Adult Human Mesenchymal Stem Cells to Treat Steroid Refractory Acute Graft Versus Host Disease. Clinicaltrials.gov NCT00366145. Registered August 17, 2006.

A Dose-escalation Safety Trial for Intrathecal Autologous Mesenchymal Stem Cell Therapy in Amyotrophic Lateral Sclerosis. Clinicaltrials.gov NCT01609283. Registered May 18, 2012.

**Electronic supplementary material:**

The online version of this article (doi:10.1186/s13287-016-0370-8) contains supplementary material, which is available to authorized users.

## Background

Regenerative medicine endeavors to surpass traditional treatments through the use of biological products to restore damaged or diseased tissues that are otherwise beyond repair. Integral to many regenerative therapeutic strategies is the use of adult stem cells, and particularly the use of mesenchymal stromal cells (MSCs) for either their regenerative or immune-regulatory properties [1]. Currently, over 246 clinical trials utilizing allogenic or autologous MSCs [2] are being performed worldwide to treat various diseases including osteoarthritis [3], atherosclerotic renal artery stenosis (ARAS) [4], multiple system atrophy (MSA) [5], graft versus host disease [6], and amyotrophic lateral sclerosis (ALS) [7]. Despite the growing number of clinical trials, there are currently no US FDA-approved MSC-based products [2]. Challenges for clinical translation of MSC-based therapies largely lie in the production and basic characterization of the MSC product [2].

Cell therapy using MSCs is largely limited by the cell harvesting and manufacturing of the MSC product. In recent years, cell therapy research has moved away from embryonic stem cells and induced pluripotent stem cells due to ethical and safety concerns over these cell types. Various alternative tissue sources of MSCs have been identified, including bone marrow, adipose tissue, umbilical cord, Wharton’s jelly, and gingival tissue. The majority of studies utilize MSCs derived from the bone marrow (BMSCs) owing to their potential to differentiate into various mesenchymal tissues, including bone, cartilage, and fat, as well as their immune-regulatory functions. However, bone marrow as a source of MSCs is limited by the invasive and painful aspiration procedure, and very low abundance of MSCs that typically account for 0.001–0.01 % of cells [8]. Adipose tissue has been identified as an MSC-rich tissue, in which 1–10 % of the stromal fraction is MSCs [9, 10], which also undergo multi-lineage differentiation in vitro [9–13]. These attributes are advantageous and also permit autologous transplantation, which is particularly important for non-fatal diseases (e.g., wound healing, osteoarthritis, or aesthetic procedures). Although the anatomical location of harvesting may have some impact on the yield of adipose-derived MSCs (AMSCs) [14, 15], variability in processing, manufacturing, and delivery of MSC/AMSCs may have larger implications on cell therapy outcomes [16].

AMSCs preparations for cell therapy vary from minimal processing (isolation of the stromal vascular fraction) to *ex vivo* expansion of the processed lipoaspirate [10]. The *ex vivo* expansion of AMSCs from the processed lipoaspirate is performed with either fetal bovine or calf serum (FBS or FCS), or under nonzoonotic conditions using human platelet lysate (hPL) [12, 17]. Previous studies have shown that culturing AMSCs in good manufacturing practices (GMP)-grade hPL provides a growth advantage, and the cellular yields were significantly greater for AMSCs grown in 5 % hPL compared to 10 % FBS or FCS [12, 17]. Tissue culture practices may also influence AMSC growth, where contact inhibition and/or cryopreservation may affect their function [18–20]. Finally, the therapeutic delivery of MSCs also varies among clinical trial protocols; MSCs are commonly cryopreserved, thawed, and administered, or allowed to recover in culture for up to 4 days prior to administration. It is currently not known how preparation procedures prior to administration may impact the function of MSCs following infusion or application.

Despite differences in isolation, production, and administration, characterization of an MSC-based product is largely limited to measuring the expression of a subset of classical cell surface markers, including CD90, CD73, CD105, and CD44, and absence of expression of CD45 or CD31 as defined by the International Society for Cellular Therapy (ISCT) and the International Federation of Adipose Therapeutics and Sciences (IFATS) [2, 11]. These markers only really serve to identify cells as MSCs so additional markers are needed to get information regarding potency and function of the cells, the differentiation potential, and how cultured cells change over time during manufacturing. To gain a better understanding of the MSC surface proteome, techniques including mass spectroscopy- and flow cytometry-based antibody screening assays have been used to characterize AMSC surface proteins and to determine the heterogeneity of MSC populations [21–26]. While these techniques are highly relevant for screening purposes, these studies have significant limitations in that they rarely utilize clinical-grade AMSCs or report whether the cells maintain homogeneity during manufacturing steps. As such, product characterization remains an unmet need for translational therapies using AMSCs. In this study, we utilized clinical-grade AMSCs grown in GMP- hPL, characterized the surface marker transcriptome of these cells, and validated the expression of five classical and nine non-classical markers.

## Methods

### Primary cell isolation and sample preparation for RNA analysis

#### Primary bone cells

Bone tissue was mechanically disrupted using a scalpel and resulting bone chips were plated onto tissue culture dishes in complete media [advanced minimum essential medium (MEM), 10 % phosphate-buffered saline (PBS), 100 U/ml penicillin, 100 g/ml streptomycin, 1x GlutaMAX] and maintained at 37 °C, 5 % CO_2_. Bone cells were plated into new culture dishes and passaged three times, at which time 1 × 10^6^ cells were harvested for RNA analysis.

#### Primary chondrocytes

Human cartilage was first digested with 0.2 % pronase in complete media [Dulbecco’s modified Eagle’s medium [DMEM]/F12 10 % FBS, 100 U/ml penicillin, 100 g/ml streptomycin, 50 μg/mL gentamycin) for 1 h at 37 °C with shaking in a cell culture incubator. Following incubation with pronase, the cartilage was washed twice with PBS, then incubated with 0.036 % collagenase-P overnight at 37 °C in a cell culture incubator. The next day, undigested cartilage was removed using a cell strainer (BD Falcon) and flow-through containing primary chondrocytes was pelleted and washed twice with PBS. Primary chondrocytes were plated onto a tissue culture plate and maintained at 37 °C, 5 % CO_2_, after which 1 × 10^6^ cells were harvested for RNA analysis.

#### Bone marrow-derived stromal cells

Primary human BMSC were purchased directly from Lonza and expanded using our previously described procedures [27]. Cells were cultured up to passage 5 and plated on standard tissue culture plastic until 70–80 % confluent for harvest.

#### Fibroblasts

Primary human gingival fibroblasts (HGF) and human periodontal ligament (HPDL) cells were established from patient gingival connective tissue explants, as previously described [28]. Cells were cultured at 37 °C in a 5 % CO_2_/95 % air atmosphere in DMEM containing 10 % FCS.

### Real-time reverse transcriptase quantitative PCR analysis

Total RNA was isolated from primary cultured cells using either Trizol® Reagent (Thermo) or miRNeasy Mini Kit (Qiagen) according to respective protocols. The SuperScript III First-Strand Synthesis System (Invitrogen) was used to reverse transcribe RNA into cDNA, which was used as a template for real-time PCR analysis. Real-time reactions were performed with 10 ng cDNA per 10 μl with the QuantiTect SYBR Green PCR Kit (Qiagen) and detected using the CFX384 Real-Time System (BioRad). Gene expression levels were normalized to the housekeeping gene, *GAPDH*, and quantified using the 2^(−delta delta Ct) method. Gene specific primers are listed in Additional file 1: Table S1.

### AMSC isolation and cell culture conditions

Mesenchymal stromal cells were derived from lipoaspirates obtained from consenting donors and clinical trial patients with approval from the Mayo Clinic Institutional Review Board (IRB) as previously described [12, 29]. After harvesting, fat tissue was digested with collagenase (type I at 0.075 %; Worthington Biochemicals) for 1.5 h at 37 °C. The stromal vascular fraction was isolated by low speed centrifugation (400 *g* for 5 min), the supernatant was removed, and the cell pellet was rinsed with PBS and passed through a cell strainer (70 μm) (BD Biosciences). Buffered ammonium solution (154 mM NH_4_Cl, 10 mM KHCO_3_, 0.1 mM EDTA) was used to lyse erythrocytes. The resulting cell fraction was plated onto tissue culture flasks in standard culture medium (advanced MEM) with 5 % hPL (Mill Creek Life Sciences), 2 mM L-glutamine (Invitrogen), and antibiotics (100 U/ml penicillin, 100 g/ml streptomycin) and maintained 37 °C in 5 % CO_2_ at a cell density of 1.0–2.5 × 10^3^ cells/cm^2^.

Prior to cryopreservation, freshly isolated AMSCs were harvested for flow cytometric analysis (pre-thaw samples), and the remaining cells were frozen in 1 ml aliquots of up to 20 × 10^6^ cells/mL with CryoStor CS10 Cryopreservation Medium (BioLife Solutions, Stem Cell Technologies) and stored in liquid nitrogen. Post-thaw samples were recovered from cryopreservation by placing the vial in a 37 °C water bath to thaw, then transferred to 10 ml of growth media and centrifuged for 5 min at 500 *g*. Cells were resuspended in growth media and approximately 1 × 10^6^ cells were harvested for flow cytometric analysis. The remaining thawed cells were plated in T-175 cm^2^ flasks at a density of up to 3000 cells/cm^2^ and cultured for 4 days at 37 °C in 5 % CO_2_. After 4 days’ culture, cells were harvested for flow cytometric analysis.

### Flow cytometry

Cells were harvested from culture, and approximately 1 × 10^6^ cells were added to tubes and centrifuged at 500 *g* for 5 min. The supernatant was removed and samples were incubated with 50 μl of mouse serum for 5 min at room temperature. Table 1 describes the antibodies and antibody mixes. Primary antibody mixes were added to respective tubes, vortexed, and incubated for 15 min at room temperature in the dark. A total of 3 ml of PBSFE [PBS with 5 mM NaEDTA (Sigma) and 1 % bovine serum albumin (Sigma)] was added to each tube and centrifuged at 500 *g* for 5 min. The supernatant was discarded and 200 μl of 1 % paraformaldehyde (Electron Microscopy Sciences) was added prior to acquisition. The flow cytometric acquisition was performed on a Beckman Coulter Gallios (Beckman Coulter). The cells were acquired using forward scatter and side scatter and gated to exclude debris. The cytometer acquired 50,000 AMSC events. The analysis was performed using Kaluza software (Beckman Coulter), where population gates were defined (%Gated) and mean fluorescence intensity (MFI) values were generated by the software based on fluorescence intensity of gated cell populations.

**Table 1:** List of antibodies used for flow cytometry validation

Antibody	Fluorophore	Company	Catalog number
CD163	FITC	Beckman Coulter	B17492
CD140B	PE	BD	558821
CD248	Alexa Fluor 647^a^	Abgent	AP6756b
CD146	PC5	Beckman Coulter	A22364
CD200	PE-Cy7	BD	562125
CD36	APC	Beckman Coulter	A87786
CD34	APC-A750	Beckman Coulter	A89309
CD44	Pac Blu	Beckman Coulter	B37789
CD44	PC7	eBioscience	25-0441
CD276	APC	eBioscience	17-2769-41
CD271	FITC	Biolegend	345104
CD105	PE	Beckman Coulter	A07414
CD73	PerCP eFluor710	eBioscience	# 46-0739-42
CD73	PE	BD	#550257
CD90	FITC	Beckman Coulter	IM1839U
CD274	PC7	Beckman Coulter	A78884
CD14	ECD	Beckman Coulter	IM2707U
HLA-ABC	APC	eBioscience	17-9983
HLA-DR	Pac Blu	Beckman Coulter	A74781
CD45	Krome Orange	Beckman Coulter	A96416

*APC* allophycocyanin, *FITC* fluorescein isothiocyanate, *PC7* R-phycoerythrin cyanin 7, *PE* R-phycoerythrin

^a^ Custom Beckman Coulter-labeled antibody

### RNA-sequencing and bioinformatic analysis

Following RNA isolation using the miRNeasy Mini Kit (Qiagen), RNA-seq was performed as previously described [13]. Briefly, RNA was prepared for sequencing using the TruSeq RNA Sample Prep Kit v2 (Illumina) and was analyzed using Illumina HiSeq 2000 with TruSeq SBS Kit v3 and HCS v2.0.12 data collection software. Sequence data were processed using MAPRSeq (v.1.2.1) and a bioinformatics workflow (TopHat 2.0.6, HTSeq, and edgeR 2.6.2), where expression data were normalized using the reads per kilobase per million (RPKM) method.

RNA-seq data were further analyzed using the DAVID Bioinformatics Resources 6.7 (https://david.ncifcrf.gov/) [30, 31]. Hierarchical clustering was performed using GENE-E (v3.0.228; Broad Institute, Cambridge, MA, USA). RNA-seq data were deposited in the public Gene Expression Omnibus (GEO) repository under the accession number [GEO:GSE84322].

### Statistical analysis

Data were analyzed using GraphPad Prism v6 (GraphPad Software) and are presented as mean and individual data points. One-way ANOVA was used for multiple comparisons and post hoc analysis using Dunn’s multiple comparisons test was used to compare pre-freeze, post-thaw, and 4-day culture, where significance was set at *p* < 0.05.

## Results

### AMSCs alter surface marker gene expression during proliferating or confluent culture conditions

**Fig. 1:**
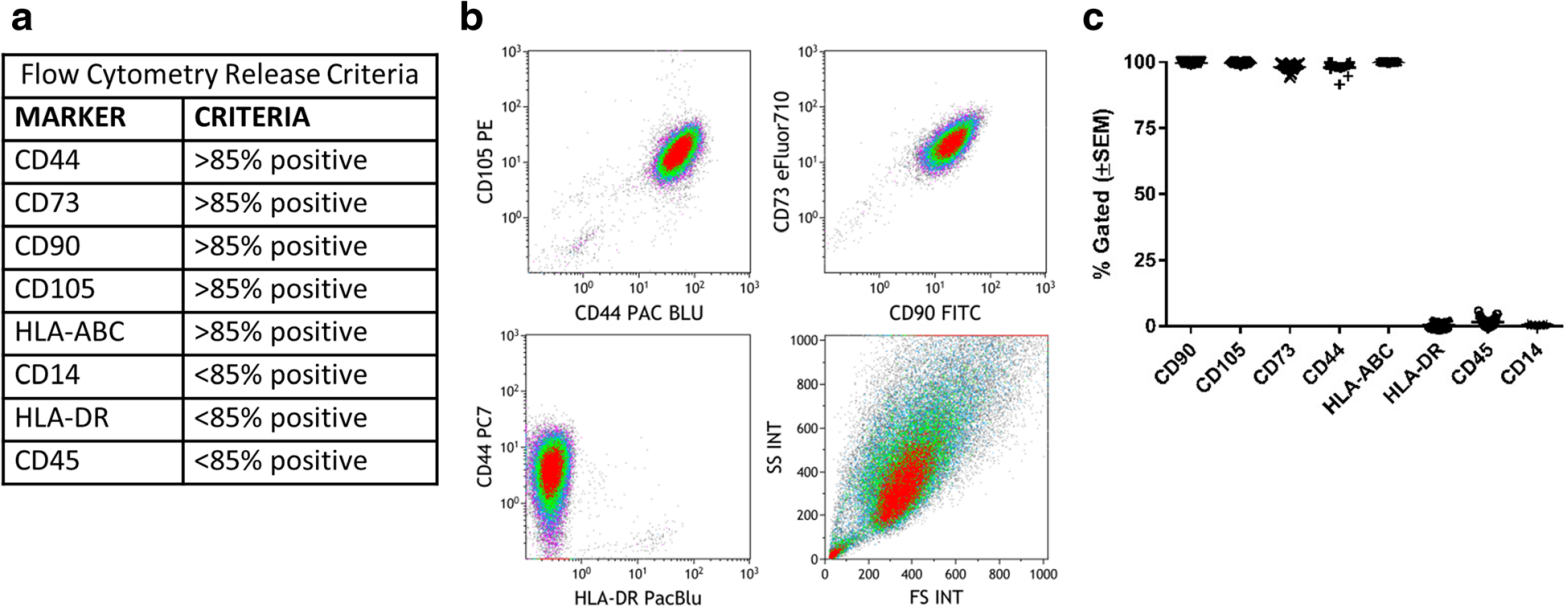
Traditional phenotyping of clinical-grade adipose-derived mesenchymal stromal cells (AMSCs) expanded in human platelet lysate. **a** Clinical-grade AMSCs grown in human platelet lysate were expanded *ex vivo* and immunophenotyped using flow cytometry according to the release criteria presented in this table. **b** Representative flow cytometry scatter plots show AMSCs are a homogeneous population of cells and exhibit surface expression of standard cell surface markers, including CD105, CD44, CD73, and CD90, and are negative for HLA-DR. **c** Analysis of the flow cytometry release criteria across clinical-grade AMSCs from 15 donors demonstrated minimal variability in the population frequency (% Gated) of the surface markers. All AMSC donor cells were >85 % positive for CD90, CD105, CD73, CD44, and HLA-ABC, and were <85 % positive for HLA-DR, CD45, and CD14

Production of clinical-grade AMSCs for cell therapy must yield a consistent product and cell surface markers are frequently used as part of the release criteria for many laboratories. The release criteria used to characterize AMSCs utilized herein are shown in Fig. 1a, where AMSCs must express classical markers including CD44, CD73, CD90, CD105, and HLA-ABC, and lack the expression of HLA-DR, CD14, and CD45 [11]. We found that the expression of these markers was homogeneous and uniform, with close to 100 % of gated cells analyzed by flow cytometry either expressing positive markers or lacking negative markers (Fig. 1b). All positive markers were consistently above 90 %, and negative markers were expressed in <5 % of gated cells across the different donors (Fig. 1c). The data presented here support the literature demonstrating that these markers are characteristic of AMSCs and are also present on clinical-grade AMSCs expanded in hPL.

### Identification of non-classical cell surface markers of AMSCs and mesenchymal cells by high-throughput quantitative PCR screening

**Fig. 2:**
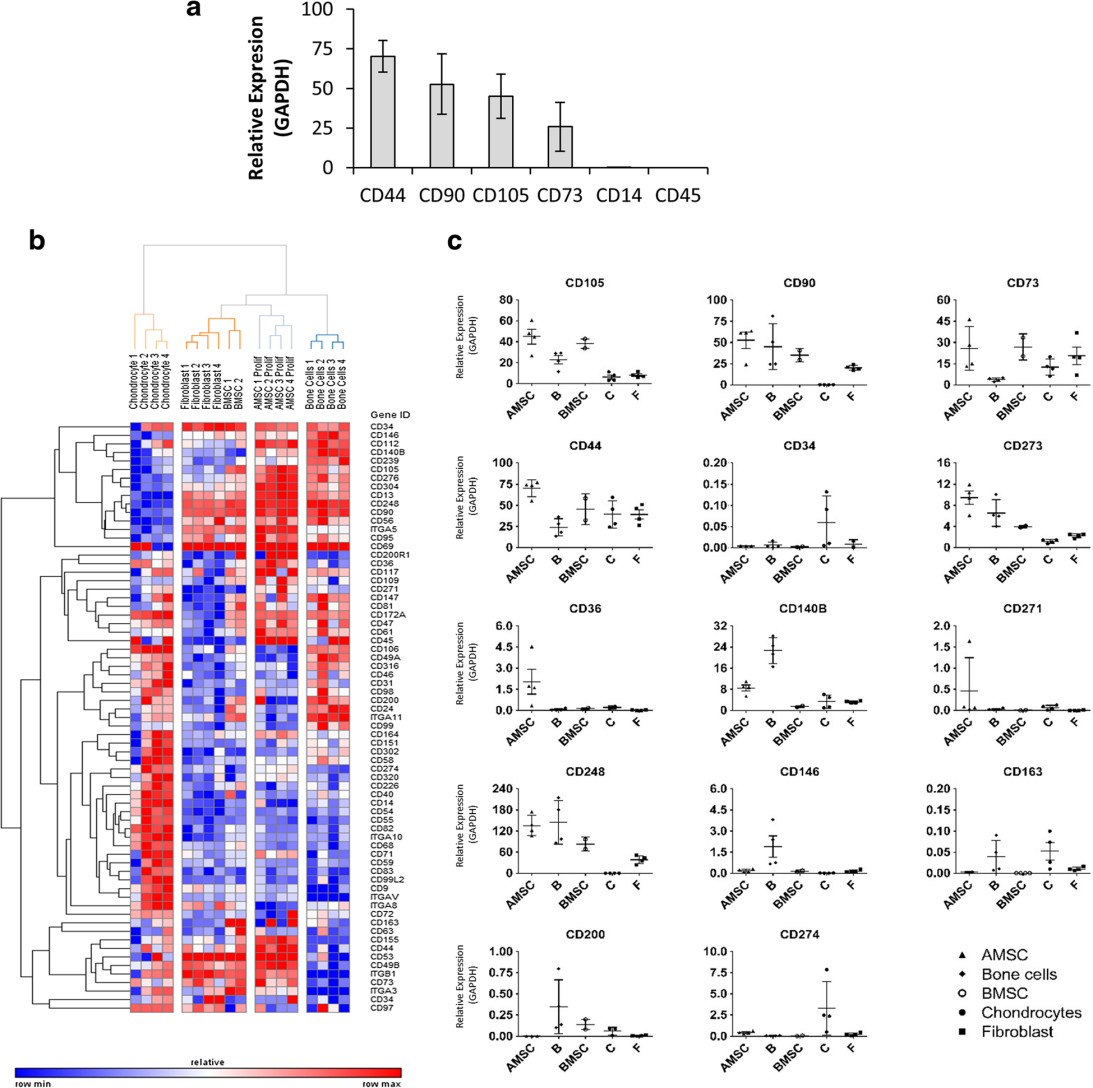
Gene expression profiling and validation of cell surface markers across multiple mesenchymal cell types. **a** Gene expression of traditional markers by adipose-derived mesenchymal stromal cells (*AMSCs*) was analyzed using quantitative PCR (qPCR). AMSCs have relatively high expression levels of CD44, CD90, CD105, and CD73, and low or no expression of CD14 and CD45. To identify AMSC specific surface markers, high-throughput qPCR screening of 69 surface markers curated from the literature was performed on various mesenchymal cell types, including AMSCs (*n* = 4), bone marrow-derived stromal cells (*BMSCs*) (*n* = 2), primary bone cells (***b***) (*n* = 4), primary chondrocytes (*C*) (*n* = 4), and primary fibroblasts (*F*) (*n* = 4). **b** Hierarchical clustering analysis of qPCR data across multiple cell types shows that AMSCs have a unique phenotype at the gene expression level. **c** Comparison of different cell types revealed that classical surface markers, including CD44, CD73, and CD90, are expressed by not only AMSCs but also other cell types. Furthermore, nine non-classical markers were selected based on differential expression between the various mesenchymal cells. These markers, together with classical markers, were used to develop a novel antibody panel to characterize AMSCs

The current AMSC cell surface markers were developed to define cell populations with tri-lineage potential and demonstrate only positive or negative characteristics. However, the expression or absence of these markers is unable to provide insight into the biological functions of AMSCs. To identify non-classical surface markers that are unique and have potential biological importance, we developed a relative quantitative real-time PCR (qPCR) panel of 69 cell surface genes based on proteomics studies of MSCs [21] and other markers from the literature. Expression of classical markers using qPCR demonstrated a higher abundance of transcripts for positive markers compared to negative markers across four donors of AMSCs (Fig. 2a), indicating that qPCR may be a surrogate for flow cytometry for classical cell surface markers.

Evaluation of the qPCR panel across four clinical-grade AMSCs donors revealed 66/69 cell surface markers were detected. Of the cell surface markers analyzed, CD248 was the most highly expressed gene and demonstrated low co-variance between donor samples (data not shown). The surface marker qPCR panel was also used to analyze surface marker expression across other mesenchymal cell types, including BMSCs, fibroblasts, bone outgrowth cells, and primary digest chondrocytes. Hierarchical clustering analysis was used to compare AMSC surface markers to other cell types and revealed distinct clusters for the different cell types. This unsupervised analysis reveals that AMSCs form a unique cluster and that these cells are most similar to fibroblasts and BMSCs (Fig. 2b). The clustering analysis demonstrates that AMSCs are a distinct cell population and may be identified using gene expression profiling techniques for a panel of cell surface markers.

To develop a novel antibody panel that can distinguish AMSCs from other mesenchymal cell types, including fibroblasts, we compared surface marker gene expression between each lineage. Amongst the genes analyzed, CD36 was highly specific to AMSCs, whereas CD140B, CD271, and CD273 were able to discriminate AMSCs from BMSCs and fibroblasts (Fig. 2c). Compared to bone cells and chondrocytes, AMSCs did not express CD163, CD146, CD200, or CD274. In addition, CD248 was the cell surface marker most abundantly expressed by AMSCs. Taken together, these nine markers were able to distinguish AMSCs from other cell types. This demonstrates that qPCR is a useful technique for identifying informative markers for developing release criteria.

### Flow cytometric validation of classical and non-classical surface markers in a cohort of clinical-grade AMSCs grown in human platelet lysate

**Fig. 3:**
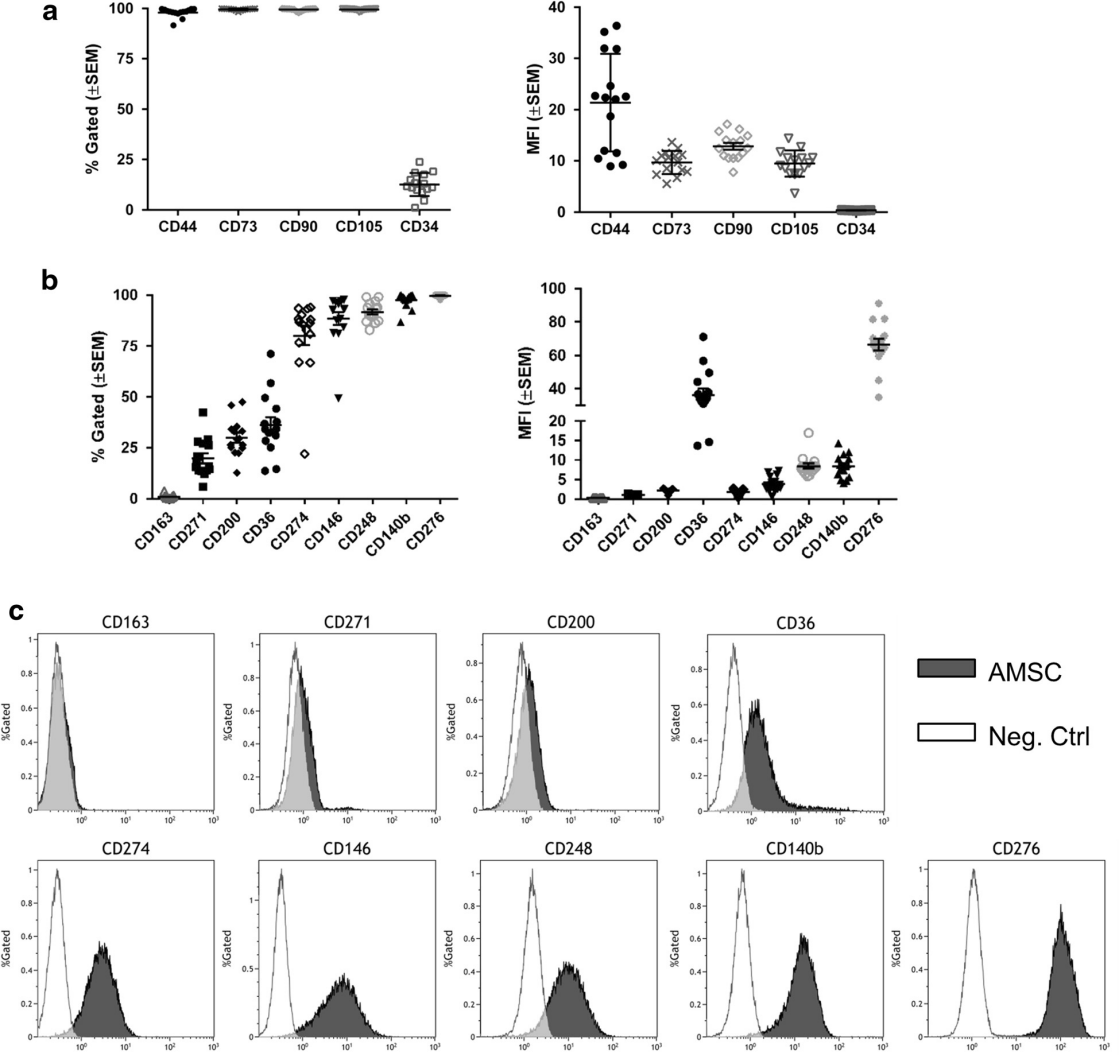
Validation of nine non-classical markers by flow cytometry among 15 clinical-grade adipose-derived mesenchymal stromal cells (*AMSCs*). Expression of five classical (**a**) and nine non-classical markers (**b**) was validated by flow cytometry across 15 additional freshly isolated and expanded AMSC donors. Surface marker expression was evaluated by the percentage of the gated cell population and mean fluorescence intensity (*MFI*). **c** Representative histograms of surface marker expression compared to unstained AMSCs (negative control). Of the non-classical markers, CD36 exhibited two cell populations which varied from patient-to-patient

Flow cytometry is the gold standard clinical tool for analyzing expression of cell surface markers and evaluating the cell population composition in a sample. We performed flow cytometry and characterized the expression of classical MSC markers (CD34, CD73, CD105, CD44, and CD90) (Fig. 3a). The surface localization of the nine non-classical markers identified through gene expression analysis (CD163, CD271, CD200, CD36, CD273, CD274, CD146, CD248, and CD140b) was also validated across 15 clinical-grade AMSCs grown in hPL before cryopreservation (Fig. 3b). Compared to classical markers, expression of each of the non-classical markers had greater donor-to-donor heterogeneity and abundance (as indicated by MFI) across all donors. Among the newly identified markers, CD276 demonstrated a similar expression pattern to the classical positive markers, with >95 % cells gated expressing this marker at robust levels (Fig. 3b). Conversely, CD163 was minimally expressed, in <4 % of gated cells across all the donors, indicating that this protein is not generally expressed on AMSCs grown in hPL. Of the new markers analyzed, CD271 and CD200 were distinguishable from negative controls; however, they were expressed at low levels among a small proportion of the population (Fig. 3b). These results suggest that the manufacturing procedure consistently isolated similar cell populations from various donors. Furthermore, these data demonstrates that eight out of nine non-classical markers are expressed on AMSCs and that CD163 is a novel negative marker of this population of cells.

**Fig. 4:**
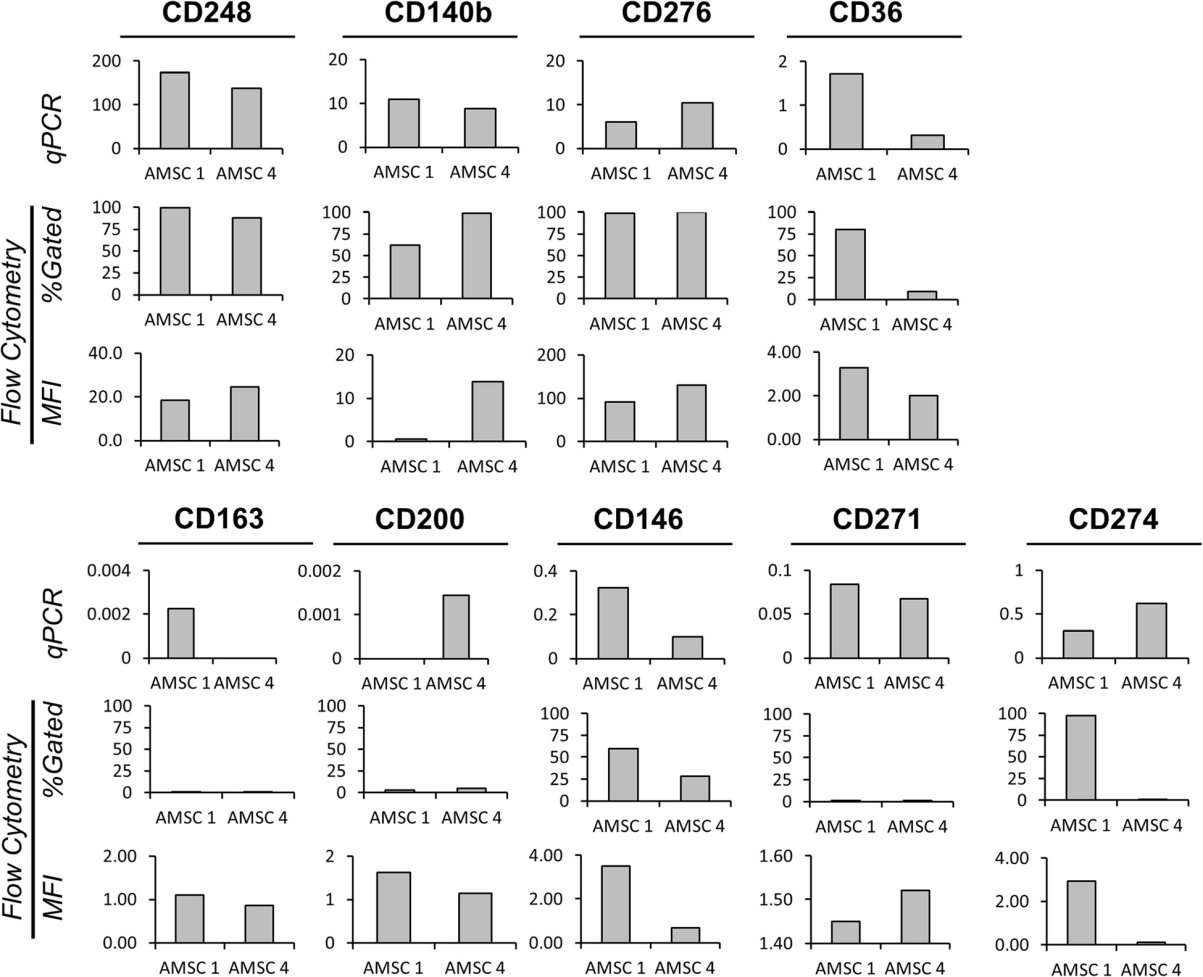
Expression of novel markers by quantitative PCR (*qPCR*) and flow cytometry. Gene expression data were compared to flow cytometry data for two donors [adipose-derived mesenchymal cell (*AMSC*) donors 1 and 4]. Highly abundant markers showed good concordance (*top panel*) between the techniques, whereas lower abundance markers showed variability (*bottom panel*). In particular, CD200 and CD274 were not correlated. *MFI* mean fluorescence intensity

Various post-transcriptional regulation mechanisms can modulate the translation of mRNA to protein, and may be a source of discrepancy in marker expression. Figure 4 provides examples of qPCR and flow cytometry data for two different AMSC donors across the non-classical markers. Markers that demonstrated similar trends between the gene expression and percentage of gated cells included CD248, CD276, CD36, CD163, CD200, CD146, and CD271. Conversely, CD140b and CD274 were not observed to show similar trends. These data suggest that, for some surface markers, mRNA expression may be a surrogate for protein expression.

### Cell surface marker expression during various manufacturing conditions

**Fig. 5:**
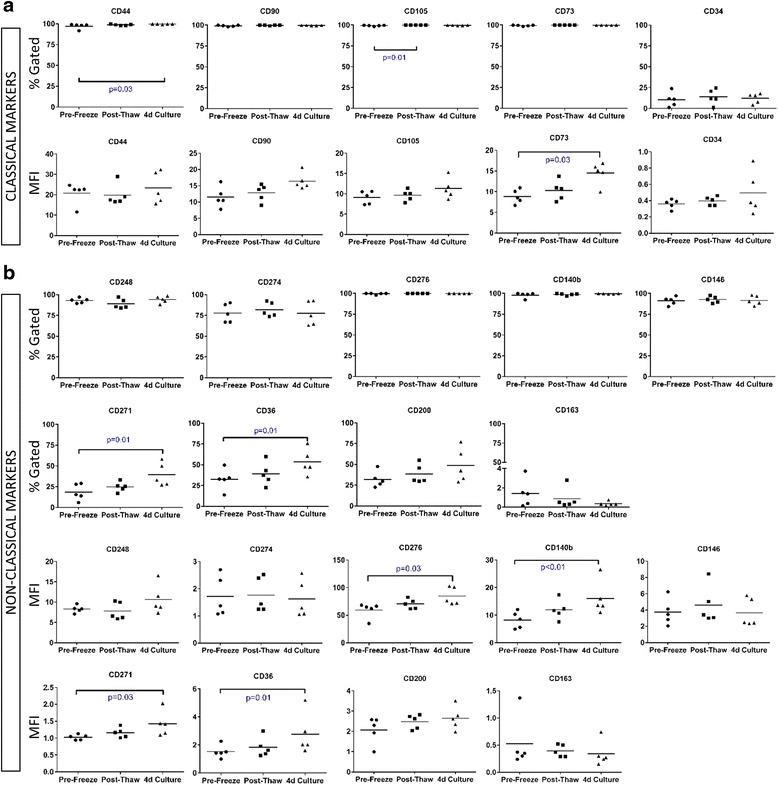
Effect of cryopreservation on surface marker expression. Flow cytometry for all 14 markers was performed on samples from 5 adipose-derived mesenchymal stromal cell (AMSC) donors before cryopreservation (pre-freeze), immediately after rescue from cryopreservation (post-thaw), and 4 days after revival from cryopreservation (4d Culture). **a** AMSCs were >90 % positive for classical surface markers across all manufacturing conditions, except CD34 which was a negative marker. **b** Non-classical surface markers exhibited variability both in population positivity (%Gated) and mean fluorescence intensity (*MFI*) as cells were processed through the various manufacturing conditions. One-way ANOVA and post-hoc testing were performed to identify variables that were statistically significant at *p* < 0.05

AMSCs are commonly administered for cell therapy by one of three methods: (i) fresh expansion; (ii) cryopreserved, thawed, and immediately administered; or (iii) cryopreserved and allowed to recover in culture for up to 4 days. To evaluate whether cryopreservation modulated cell surface marker expression, we performed flow cytometric analysis on samples from five donors at the aforementioned clinically relevant time points (Fig. 5). Classical markers CD44, CD90, CD105, and CD73 were expressed by >90 % of gated cells across all three time points (Fig. 5a). Among these markers, CD105 and CD44 demonstrated a statistically significant increase in gated cells following either cryopreservation or 4 days in culture. The MFI for CD44 and CD105 also showed a general trend for increased expression (Fig. 5a). CD73 fluorescence intensity was also significantly increased compared to other classical markers, indicating a potential effect of cryopreservation or adaptation to the culture environment. These data suggest that classical markers are constitutively expressed irrespective of manufacturing conditions.

We also evaluated the expression of non-classical markers across the three manufacturing time points (Fig. 5b). CD276 and CD140b were observed to exhibit a similar expression pattern to classical markers, as >90 % of gated cells were positive for these markers and were unchanged over the different conditions. Furthermore, the MFIs for these markers were significantly upregulated by day four after recovery from cryopreservation (Fig. 5b), which was also observed for the classical marker CD73. However, the non-classical markers generally demonstrated greater donor-to-donor variability compared to classical markers (Fig. 5b). In particular, CD271 and CD36 were the only markers analyzed that demonstrated a >20 % increase in the percentage of positive cells by day four after thawing, whereas CD44 and CD105 only increased by 2.43 % and 0.34 % respectively. The increased percentage of positive cells for CD271 and CD36 was also corroborated by a significant increase in MFI (Fig. 5b). Further studies are required to determine the biological importance of the increased abundance of CD271- and CD36-positive cells, but it may indicate positive selection of more robust cells or that recovery from cryopreservation is necessary for expression of growth factor receptors (CD271/NGFR) and fatty acid transporters (CD36). Collectively, these results suggest that cryopreservation does not modulate the expression of classical and non-classical cell surface markers. However, recovery in culture may allow optimal surface level expression of these proteins.

### High-resolution surface marker gene expression analysis of clinical-grade AMSCs grown in human platelet lysate

During the manufacturing process AMSCs are expanded *ex vivo* to produce the large number of cells required for therapeutic applications. Throughout the process it is important to monitor the growth of the AMSCs and particularly the confluence of the cultures (surface area covered by cells), as these cells experience contact inhibition. To evaluate whether cell surface marker gene expression was modulated by the confluent state of the culture, RNA from proliferating (70–80 % confluent) and confluent (100 % confluent) cultures was analyzed. Gene expression analysis using the high-throughput qPCR panel developed above revealed 29 genes that were up-regulated greater than 2-fold in confluent cells and nine genes that were 2-fold up-regulated in proliferating cells. Classical surface markers CD44, CD105, and CD73 were not modulated by confluency; however, both CD90 and CD34 were >2-fold up-regulated in confluent cultures. Together, these results indicate that cell surface markers are directly modulated by the tissue culture conditions.

**Fig. 6:**
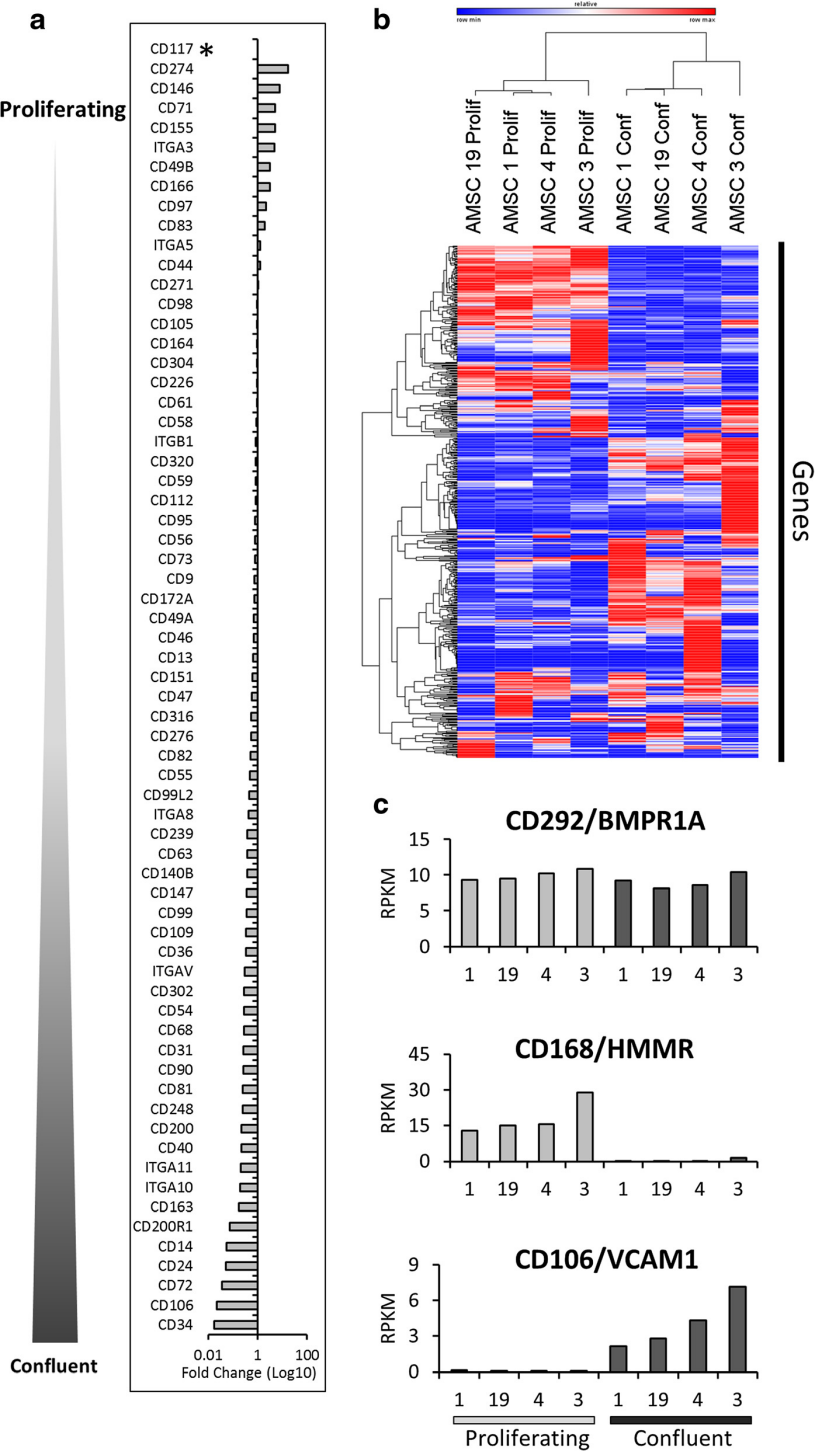
High-resolution RNA-sequencing (RNA-seq) analysis of surface marker gene expression by proliferating and confluent adipose-derived mesenchymal stromal cells (*AMSCs*). **a** Gene expression profiling using quantitative PCR for 69 cell surface protein-encoding genes reveals some surface markers are differentially expressed between proliferating (~70–80 % confluent) and confluent (100 % confluent) AMSCs. Values indicate fold-change (Log10 transformed) of confluent over proliferating, and are averages of samples from four different AMSC donors. Fold-change analysis shows markers that are differentially expressed between proliferating and confluent cultures. To further evaluate differential surface marker expression, RNA-seq was performed on proliferating and confluent AMSCs from four different donors. **b** Expression values for 551 cell surface genes expressed at a magnitude >0 reads per kilobase per million (*RPKM*) by all AMSC donors were extracted from the RNA-seq data set and subjected to hierarchical clustering analysis, which revealed distinct expression patterns for proliferating and confluent cells. **c** Representative graphs of genes derived from RNA-seq analysis shows CD292/BMPR1A was constitutively expressed, CD168/HMMR was only expressed by proliferating cells, and CD106/VCAM1 was only expressed in confluent cells

Recently, high-resolution gene expression analysis using RNA-seq technology was used to characterize AMSCs inherent proliferative and differentiation potential [13]. Following our observations from qPCR profiling of cell surface genes, we utilized both previously published [13] and new RNA-seq data to evaluate the expression of 707 genes encoding CD markers and related cell surface proteins as curated based on gene ontology terms for four AMSC donors. It should be noted that the RNA-seq data represented proliferating and confluent samples as indicated by the expression of cell cycle genes (Additional file 2: Figure S1). Genes that were not expressed (0 RPKM) across all samples were filtered out, which revealed that AMSCs express 551/707 surface protein-encoding genes. Furthermore, RNA-seq data also supported our initial qPCR findings, in which surface markers expressed by AMSCs showed distinct clustering patterns of proliferating and confluent samples (Fig. 6b, d, c).

To further characterize the AMSC surface transcriptome, we filtered the 551 expressed surface protein-encoding genes for expression >0.1 RPKM, and developed gene lists for the constitutive, proliferating, or confluent state. The gene lists were subjected to functional annotation analysis using the public database DAVID 6.7 [30, 31] (Table 2). Due to the selection of only cell surface protein-encoding genes as the input, “Membrane,” “Signal,” and “Plasma membrane” were commonly ranked among the highly enriched categories. However, the categories enriched in the constitutive group provide insight into the functions of AMSCs, including “Signal Transduction,” “Angiogenesis,” and “Immune response.” In the proliferating group of genes, categories including “Integrin,” “ECM-receptor interaction,” and “Biosynthetic process” suggest these cells up-regulate proteins involved in movement and replication. Conversely, the group of genes characteristic of the confluent state indicate processes involved in paracrine signaling, including “Vesicle-mediated transport” and “Regulation of transcription.” Together, these data suggest that AMSCs express different levels of mRNAs for CD markers in proliferative and confluent states.

**Table 2:** Top 25 categories from a DAVID 6.7 analysis of cell surface proteins expressed on adipose-derived mesenchymal stromal cells

	Constitutive	Proliferating	Confluent
	(*n* = 142, *p* > 0.05)	(*n* = 104, up >1.4-fold, *p* < 0.05)	(*n* = 79, up >1.4-fold, *p* < 0.05)
Rank	Category (enrichment score)	Category (enrichment score)	Category (enrichment score)
1	Membrane (48.26)	Signal (28.07)	Membrane (24.12)
2	Signal (47.47)	Membrane (27.82)	Signal (18.65)
3	Plasma membrane (28.33)	Cell adhesion (14.15)	Plasma membrane (11.82)
4	Cell adhesion (9.99)	Plasma membrane (12.38)	Tetraspanin (3.97)
5	Signal transduction (8.17)	Immunoglobulin-like (6.25)	Protease/peptidase (3.95)
6	Tetraspanin (7.20)	Tetraspanin (5.88)	ABC transporter (3.93)
7	Lipoprotein (5.52)	Integrin (5.75)	Growth factor binding (3.82)
8	Angiogenesis (5.11)	Response to wounding (4.85)	Cell adhesion (3.39)
9	B cell activation/Fas pathway (5.08)	Immunoglobulin-like V set (4.57)	Secreted (3.30)
10	Cell migration (4.86)	Metalloprotease (4.47)	Semaphorin (2.95)
11	Apoptosis (4.68)	Cell–cell adhesion (4.37)	Immunoglobulin-like (2.95)
12	Semaphorin (4.63)	Transferase (3.71)	Immune cell activation (2.55)
13	Immune response (4.38)	Semaphorin/integrin (3.56)	Vesicle-mediated transport (2.40)
14	Calcium-mediated signaling (4.20)	Transport (3.40)	Cytokine–cytokine receptor interaction (2.33)
15	Cytokine binding (4.12)	Extracellular matrix-receptor interaction (3.37)	Cell migration (2.29)
16	Activation immune response (4.00)	Cell motion (3.36)	Integrin (2.24)
17	Cell motion (3.68)	Differentiation (3.04)	Glycoprotein metabolic process (2.07)
18	Protein kinase cascade (3.64)	Epidermal growth factor (2.88)	Cell proliferation (1.96)
19	Integrin (3.31)	Low-density lipoprotein (2.32)	Membrane fraction (1.93)
20	Tyrosine protein kinase (3.04)	Biosynthetic process (2.20)	Immunoglobulin V-set (1.84)
21	Immune cell activation (3.02)	Secreted (2.13)	Regulation of transcription (1.56)
22	Signal-anchor (3.01)	Immune response (2.11)	Response to wounding (1.48)
23	Low density lipoprotein receptor (2.91)	Membrane fraction (2.04)	Regulation of immune activation (1.38)
24	Stress-activated protein kinase (2.90)	Magnesium ion binding (1.92)	Protein kinase cascade (1.18)
25	Wnt receptor pathway (2.88)	Cytokine binding (1.75)	Carbohydrate binding (1.07)

## Discussion

Numerous clinical trials are currently being performed using AMSCs as a cellular therapy for various diseases worldwide [32, 33]. Standardization of the production procedures and accurate characterization of the MSC product to ensure patient safety has been a significant concern for regulatory agencies governing the approval of biological license applications [2]. Our study identified and validated the expression of 14 classical and non-classical surface markers on clinical-grade AMSCs expanded in hPL adherent to good manufacturing practices (GMP-hPL). Furthermore, we evaluated surface marker expression during processes for preparing cells for clinical administration and demonstrated variability of these markers with doublings/day and cryopreservation.

Traditionally AMSCs have been expanded with FBS as part of the culture media to provide growth factors and other proteins to support proliferation. However, potential zoonotic pathogens and immunogenic reactions from FBS are concerning for the clinical administration of MSCs, which led to the development of nonzoonotic substitutes including hPL [12, 34]. As part of the production of clinical-grade AMSCs used in these studies, we expanded our MSC product in GMP-hPL, which has previously been shown to support proliferation and genomic stability [12]. Furthermore, hPL contains proteins important for healing, including FGF/EGF, TGF-β/BMP, and VEGF/PDGF, which may facilitate AMSC growth and stability [35]. However, due to the differences in composition of FBS and hPL, including cytokines and growth factors, there exists the potential for the selection of different adherent cell populations.

An AMSC cell population is characterized as one that adheres to plastic, expresses characteristic surface markers, and has tri-lineage potential [11]. Establishing these criteria was an important step forward for standardization of stem cell science and industry. This study validated that clinical-grade AMSCs from 15 different donors met these established criteria and also expressed a unique set of non-classical surface markers. The AMSCs utilized in this investigation were cultured in GMP-hPL, adhered to plastic, and uniformly expressed the classical surface markers CD44, CD73, CD90, and CD105 and did not express CD34. Although these markers uniformly define AMSCs, ours and other studies have observed that these markers are unable to distinguish donor differences, including variability in proliferation or trophic activity [36]. Therefore, there is an increased need to identify additional markers that not only define AMSCs but also have the potential to capture biological and manufacturing variability, as well as clinical performance.

Beyond serving as markers for cell characterization, surface proteins carry out important biological functions and are critical for cell-to-cell contact, extracellular matrix interactions, signal transduction, and transportation of molecules across the plasma membrane. Our study examined the expression of all plasma membrane protein-encoding genes (including CD markers, receptors, integrins, and transporters) by RNA-seq and identified 551/707 genes that were expressed on AMSCs. Previous studies of AMSCs and BMSCs evaluated the expression of 200–242 surface markers using mass spectroscopy or BD Lyoplate technology with flow cytometry techniques [21–24]. Together, our and others’ studies have identified and characterized a finite number of surface proteins present on the AMSC cell surface under standard conditions. A comparison of surface marker expression by qPCR expression and flow cytometry showed partial concordance, with seven out of nine markers showing similar trends (Fig. 4). Our results show that gene expression and flow cytometry techniques can be used to identify novel cell surface markers in AMSC populations. However, confirmation of cell surface proteins by flow cytometry is still necessary to confirm cell surface marker expression, as it is the primary technique used in the clinical setting.

Our data also reveal differential expression of mRNAs for CD markers in proliferative and post-proliferative AMSCs. Previous studies have also characterized the gene expression effects of confluence and doubling times on AMSCs and BMSCs [13, 37]. These studies and ours suggest that culture conditions, including proliferation state and population doublings, may affect the differentiation potential of AMSCs. In particular, mRNA expression of some surface markers is restricted to either the proliferating or confluent state (Fig. 6c). However, further studies are required to determine whether manufacturing conditions (e.g., length of culture and growth rate), as well as biological factors (e.g., donor age and disease status) would impact therapeutic potential. Our studies indicate that at least some variation in cell surface expression may emerge during AMSC production depending on the growth rate of the cell population.

Currently there is no standardization of release criteria for MSC products, and studies that define their criteria usually include the markers described by the ISCT and IFATS [2, 38]. Through the use of gene expression profiling techniques and flow cytometry, our study identified and validated nine non-classical markers that may help further characterize hPL-expanded AMSCs and improve current release criteria. In our study we validated the expression of CD163, CD271, CD200, CD36, CD274, CD146, CD248, CD140B, and CD276. These markers are also expressed on FBS-expanded AMSCs [22–25, 39–42]. Our study also identified CD163, a monocyte and macrophage marker, as a negative AMSC marker, which may be useful for characterizing clinical-grade MSC populations [43].

We also described the expression of the known immune-regulatory markers CD274 (B7H1/PD-L1) and CD276 (B7H3) on hPL-expanded AMSCs. Traditionally, mature dendritic cells produce soluble CD274 and CD276 [44]. However, the current results demonstrate that AMSCs grown in hPL also express these markers on the cell surface. Our results show that CD276 is highly expressed and may be expressed ubiquitously with other traditional markers such as CD73, CD105, and CD90. Similarly, CD274 is highly expressed, but shows greater variability between donors. The functional role of CD274 and CD276 on AMSCs has yet to be characterized; however, CD274-positive BMSCs have been shown to regulate T-cell proliferation and Th17 polarization [45, 46]. Furthermore, recent studies have shown that interferon gamma (IFNγ) priming or licensing of BMSCs may also up-regulate CD274 and enhance MSC-mediated T-cell inhibition [47, 48]. However, the function of CD276 remains controversial as this molecule may act as a co-stimulatory molecule for T-cell activation and selectively stimulates the production of IFNγ [49], or may inhibit T-cell proliferation [50]. CD274 and CD276 have the potential to serve as predictive clinical markers for MSC immunomodulatory activity. Isolation and characterization of CD274-positive and CD276-positive cells may determine whether these cells represent distinct subpopulations with enhanced immune-regulatory effects. Further studies correlating these markers with patient outcomes in clinical trials would also help to elucidate the role of these markers in hPL-expanded AMSCs.

Storage and administration of the MSC product for cell therapy may depend on the disease and the institutional infrastructure. Current clinical trials administer MSC products either without cryopreservation, cryopreserved and thawed, or allowed to recover for 4 days in culture. Previous studies have demonstrated reduced immunosuppressive properties of MSCs immediately thawed after cryopreservation, and that these properties were restored as early as 24 h after placing in tissue culture [51]. Samples from three of our five donors analyzed showed a slight decrease in CD248 expression between pre-freeze and post-thaw samples. The decrease in surface marker expression could be attributed to damage to the cell surface of the protein that reduces antibody-binding efficiency or, potentially, the sensitivity of CD248 expression to the metabolic state of the cell. We also observed a significant increase in CD105 expression between pre-freeze and post-thaw samples, as well as a significant increase in surface marker expression over 4 days for CD271, CD36, and, to a lesser extent, CD44. These results support the work of Francois and colleagues [51], whereby the recovery of AMSCs in culture for up to 4 days can result in maximal surface marker expression. Together, these data suggest that surface marker expression is modulated during the cryopreservation process and that it may be important for cell function to allow cells to recover for up to 4 days.

## Conclusions

We evaluated the expression of classical and non-classical surface markers across a cohort of 15 donors from various disease backgrounds. Our results demonstrate that our manufacturing procedures consistently produce an AMSC population that uniformly expresses classical MSC surface markers. We also characterized the expression of nine non-classical surface markers that may be used to further characterize the AMSC product. The known immunomodulatory markers CD274 and CD276 are also highly expressed on the surface of AMSCs and may be able to predict their immunomodulatory activity and clinical efficacy. Future clinical trials will help us to determine which surface markers are the best predictors of clinical outcomes for patients.

## Abbreviations

ALS, Amyotrophic lateral sclerosis; AMSC, Adipose derived mesenchymal stromal cell; ARAS , Atherosclerotic renal artery stenosis; BMSC, Bone marrow derived mesenchymal stromal cell; DMEM, Dulbecco’s Modified Eagle’s Medium; FBS, Fetal bovine serum; FCS, Fetal calf serum; FDA, Food and drug administration; Geo, Geometric Mean; GMP, Good manufacturing practices; HGF, human gingival fibroblasts; HPDL, human periodontal ligament; hPL, Human platelet lysate; IFATS, International Federation of Adipose Therapeutics and Sciences; IRB, Institutional Review Board; ISCT, International Society for Cellular Therapy; MFI, Mean fluorescence intensity; MSA, Multiple systems atrophy; MSC, Mesenchymal stromal cell; PBS, phosphate-buffered saline; qPCR, quantitative polymerase chain reaction; RNA-seq, RNA sequencing; RPKM, Reads per kilobase per million mapped reads

## Additional files

Additional file 1: Table S1. Oligonucleotide primer sequences used in qPCR reactions for the detection of CD marker gene transcript abundance. (XLSX 17.6 kb) Additional file 2: Figure S1. Representative graphs of cell cycle genes derived from RNA-seq data for AMSCs under proliferating and confluent conditions. (TIF 340 kb)
