# Supplementary figures and images for: Identification and validation of multiple cell surface markers of clinical-grade adipose-derived mesenchymal stromal cells as novel release criteria for good manufacturing practice-compliant production
Source: Stem Cell Res Ther. 2016 Aug 11;7:107. doi: 10.1186/s13287-016-0370-8 (PMC4982273; doi:10.1186/s13287-016-0370-8)

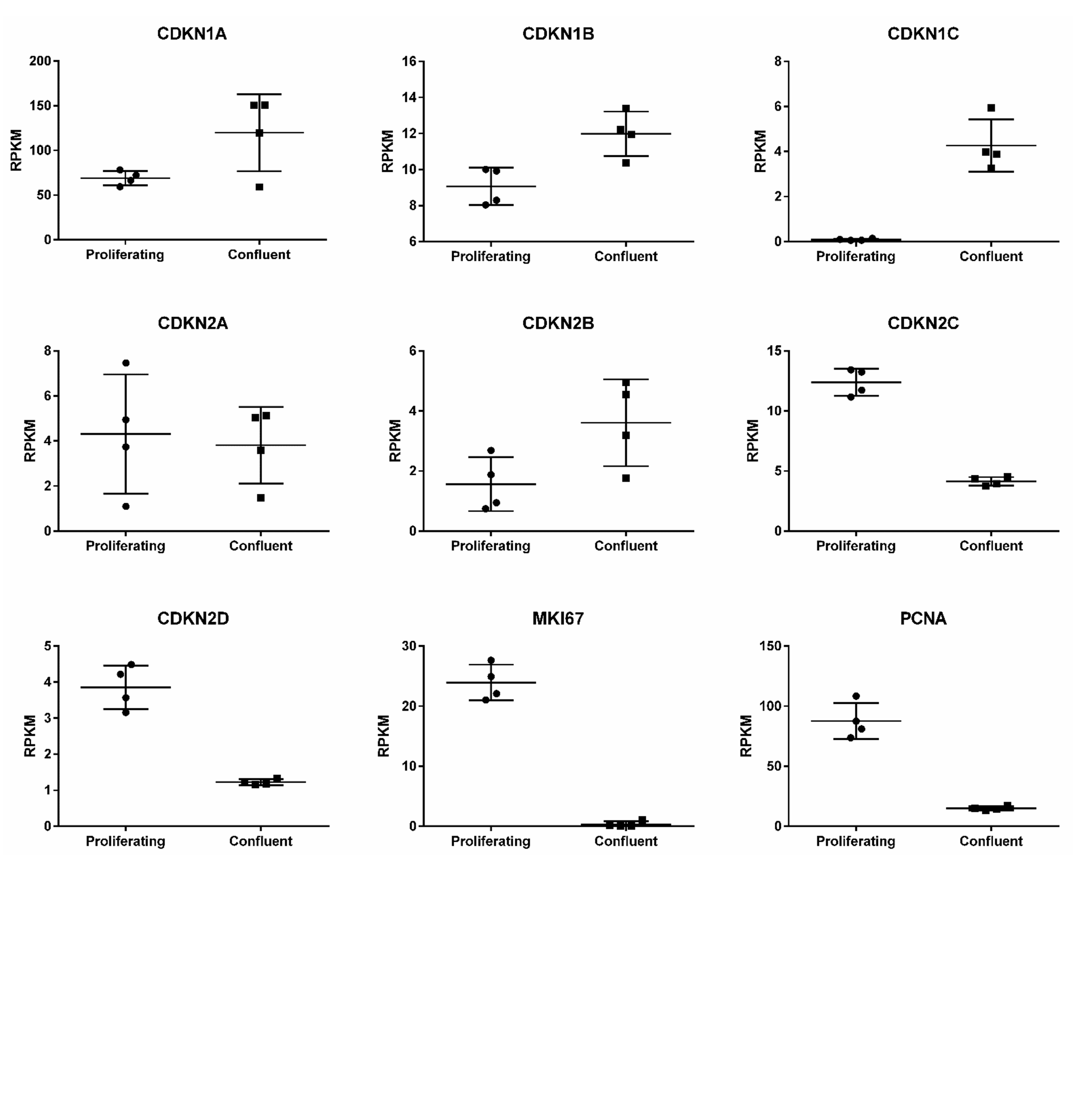

Supplement: Additional file 2: Figure S1. — Representative graphs of cell cycle genes derived from RNA-seq data for AMSCs under proliferating and confluent conditions. (TIF 340 kb) [file 13287_2016_370_MOESM2_ESM.tif]
